# PKA and PKC Modulators Affect Ion Channel Function and Internalization of Recombinant Alpha1 and Alpha1-Beta Glycine Receptors

**DOI:** 10.3389/fnmol.2018.00154

**Published:** 2018-05-14

**Authors:** Ulrike Breitinger, Lamiaa M. Bahnassawy, Dieter Janzen, Vera Roemer, Cord-Michael Becker, Carmen Villmann, Hans-Georg Breitinger

**Affiliations:** ^1^Department of Biochemistry, German University in Cairo, New Cairo, Egypt; ^2^Institute for Clinical Neurobiology, Julius-Maximilians University Würzburg, Würzburg, Germany; ^3^Department of Biochemistry, Institute of Biochemistry, Friedrich-Alexander-University Erlangen-Nürnberg, Erlangen, Germany

**Keywords:** glycine receptor, PKA, PKC, activators/inhibitors of phosphorylation, whole-cell currents, modulation kinetics, receptor internalization

## Abstract

Glycine receptors (GlyRs) are important mediators of fast inhibitory neurotransmission in the mammalian central nervous system. Their function is controlled by multiple cellular mechanisms, including intracellular regulatory processes. Modulation of GlyR function by protein kinases has been reported for many cell types, involving different techniques, and often yielding contradictory results. Here, we studied the effects of protein kinase C (PKC) and cAMP-dependent protein kinase A (PKA) on glycine induced currents in HEK293 cells expressing human homomeric α1 and heteromeric α1-β GlyRs using whole-cell patch clamp techniques as well as internalization assays. In whole-cell patch-clamp measurements, modulators were applied in the intracellular buffer at concentrations between 0.1 μM and 0.5 μM. EC_50_ of glycine increased upon application of the protein kinase activators Forskolin and phorbol-12-myristate-13-acetate (PMA) but decreased in the presence of the PKC inhibitor Staurosporine aglycon and the PKA inhibitor H-89. Desensitization of recombinant α1 receptors was significantly increased in the presence of Forskolin. Staurosporine aglycon, on the other hand decreased desensitization of heteromeric α1-β GlyRs. The time course of receptor activation was determined for homomeric α1 receptors and revealed two simultaneous effects: cells showed a decrease of EC_50_ after 3–6 min of establishing whole-cell configuration. This effect was independent of protein kinase modulators. All modulators of PKA and PKC, however, produced an additional shift of EC_50_, which overlay and eventually exceeded the cells intrinsic variation of EC_50_. The effect of kinase activators was abolished if the corresponding inhibitors were co-applied, consistent with PKA and PKC directly mediating the modulation of GlyR function. Direct effects of PKA- and PKC-modulators on receptor expression on transfected HEK cells were monitored within 15 min of drug application, showing a significant increase of receptor internalization with PKA and PKC activators, while the corresponding inhibitors had no significant effect on receptor surface expression or internalization. Our results confirm the observation that phosphorylation via PKA and PKC has a direct effect on the GlyR ion channel complex and plays an important role in the fine-tuning of glycinergic signaling.

## Introduction

**Figure 1:**
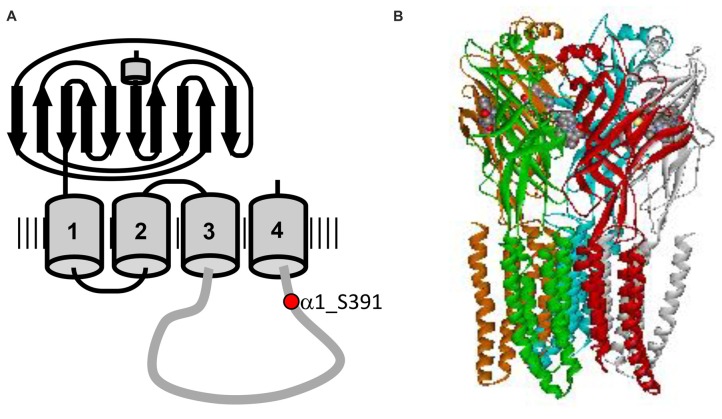
Structure of glycine receptor (GlyR). **(A)** Topology of the GlyR α1 subunit showing the long extracellular domain, four transmembrane domains TM1–4 and the long intracellular loop connecting TM3 and TM4. The protein kinase C (PKC) consensus phosphorylation sequence is located in the intracellular TM3–4 loop, with residue Ser391 marked in red. **(B)** Electron cryomicroscopy structure of GlyR α1, adapted from Du et al. (2015). Unfortunately, the structures available do not include the flexible TM3–4 loop.

Neuronal glycine receptors (GlyRs), together with GABA_A_ receptors, mediate fast inhibitory neurotransmission in the mature central nervous system (Breitinger and Becker, 2002). GlyRs mediate chloride influx that causes hyperpolarization of the postsynaptic cell, resulting in an inhibitory postsynaptic potential (Dutertre et al., 2012). Native GlyRs are composed of two different subunits GlyR α (48 kDa) and β (58 kDa). Four different α subunits, α1-α4 and one β subunit are known to date (Breitinger, 2014), encoded by their respective genes *GLRA1–4* and *GLRB* (Matzenbach et al., 1994). In addition, α-subunits can form functional homomeric ion channels while β-subunits are thought to be responsible of synaptic anchoring without having ion-channel function of their own (Breitinger and Becker, 2002; Grudzinska et al., 2005). Each subunit consists of a large N-terminal extracellular domain, four transmembrane segments (TM1–4), a long intracellular loop that connects TM3 and TM4 (TM3–4), as well as a short extracellular C-terminus (Breitinger, 2014; Figure 1). The intracellular loop linking the TM3–4 domains consists of about 100 residues and exhibits the highest degree of sequence variability among the GlyR family. It contains sites involved in receptor modulation and interaction with intracellular proteins and cytoskeletal structures (Breitinger and Becker, 2002), including potential targeting sites for protein kinases and/or phosphatases (Smart, 1997). Regulation of GlyR function by phosphorylation has indeed been observed, including altering channel properties, receptor expression, mobility or localization (Ruiz-Gómez et al., 1991; Vaello et al., 1994; Gentet and Clements, 2002; Velázquez-Flores and Salceda, 2011; Huang et al., 2015; Langlhofer and Villmann, 2016). The two most important kinases involved in phosphorylation of ion channel receptors are protein kinase C (PKC) and cAMP-dependent protein kinase A (PKA). The specific phosphorylation sites and the resulting effects on GlyR function have been explored in different cell types using different techniques and often yielded inconsistent and even contradictory results.

Here, we studied the effects of PKA and PKC modulators on inhibitory GlyRs expressed in HEK293 cells. Stimulation of PKA and PKC by Forskolin and phorbol-12-myristate-13-acetate (PMA), respectively, gave higher EC_50_ values of glycine, while inhibition of both kinases using H-89 (PKA) and Staurosporine aglycon (PKC) led to a decrease of EC_50_. Effects of kinase modulators were concentration-dependent and developed fully within 5–10 min after intracellular application. None of the kinase modulators had an immediate effect on maximum current responses. However, When the effects of PKA- and PKC activators was followed over 10 min, a significant reduction of I_max_ was observed compared to controls, while I_max_ was slightly increased relative to control in presence of kinase inhibitors. PKA modulators had no effect on GlyR trafficking, while stimulation of PKC by PMA accelerated receptor internalization and lowered cell surface expression of recombinant GlyRs. The opposite was observed with the PKC-inhibitor Staurosporine aglycon. Thus, phosphorylation was confirmed to be a specific and relevant modulatory element of GlyR activity.

## Materials and Methods

### Cell Culture and Transfection

HEK293 cells were grown in 10 cm tissue culture Petri dishes in Eagle minimal essential medium (Sigma-Aldrich Chemie GmbH, Munich, Germany) supplemented with 10% FBS (Invitrogen, Karlsruhe, Germany) and penicillin/streptomycin (Sigma-Aldrich Chemie GmbH, Munich, Germany) at 5% CO_2_ and 37°C in a water saturated atmosphere. For electrophysiological experiments, cells were plated on poly-L lysine treated glass coverslips in 6 cm dishes. Transfection was performed 1 day after cell passage using Gen-Carrier (Epoch Lifesciences, Sugarland, TX, USA): 1.3 μg of receptor DNA, 1.3 μg of green fluorescent protein DNA, and 2.6 μl GenCarrier were used, following the manufacturer’s instructions. Measurements were performed 2–4 days after transfection. HEK293 cells used for protein expression and internalization analysis were transfected using a modified calcium phosphate precipitation method. In brief, a mixture of plasmid DNA, CaCl_2_ (2.5M), 0.1× TE buffer and 2× HBS (50 mM HEPES, 12 mM glucose, 10 mM KCl, 280 mM NaCl, 1.5 mM Na_2_HPO_4_) was applied onto the cells for 6 h. A GlyR α1 to GlyR β ratio of 1:2 was used for co-expression. Transfection medium was replaced by fresh medium after 6 h. Immunocytochemical stainings were always done 24 h after transfection, biotinylation experiments were performed 48 h after transfection.

### Receptor Biotinylation

For biotinylation, transfected HEK293 cells transiently expressing the GlyR α1 or co-expressing GlyR α1-β were incubated with PKA or PKC modulators for 16 h. Surface proteins were labeled with 1 mg/ml EZ-Link Sulfo-NHS-LC-Biotin (sulfosuccinimidyl-6-[biotin-amido] hexanoate; Thermo Fisher Scientific, Waltham, MA, USA). After cell lysis, biotin-labeled protein was removed using streptavidin agarose beads (Thermo Fisher Scientific, Waltham, MA, USA). Total protein, surface protein and intracellular proteins were subjected to SDS-PAGE and Western Blotting.

### Immunostaining of Western Blots

For protein separation, 11% polyacrylamide gels were used. Gels were run at 150 V for 90 min. Proteins were transferred to a nitrocellulose membrane (GE Healthcare, Freiburg, Germany) using a wet blot transfer system (Bio-Rad, Hercules, CA, USA). For GlyR protein transfer, 2 h at 200 mA were used. Cadherin transfer required overnight blotting at 100 mA. Membranes were blocked for 1 h with 5% BSA in TBS-T (TBS with 1% Tween 20). Primary antibodies (pan-α antibody for GlyRs, MAb4a, Synaptic Systems, Göttingen, Germany, 1:500 in TBS-T and pan-cadherin, Cell Signaling Technology, Danvers, MA, USA, 1:1.500 in TBS-T) were incubated overnight at 4°C. Signals were detected using SuperSignal West Pico (Thermo Fisher Scientific, Waltham, MA, USA).

### Receptor Internalization

To stain internalized GlyRs, GlyR α1 was cotransfected with GlyR β into HEK293 cells grown on glass cover slips. Internalization experiments were performed 24 h post-transfection. Live cells co-expressing GlyR α1-β receptors were labeled with the α1-specific monoclonal antibody Mab2b (1:500 in Earle’s minimal essential medium for 1 h at 4°C). Earle’s minimal essential medium was supplemented with 10% fetal calf serum, 200 mM GlutaMAX, 100 mM sodium pyruvate, and 50 U/ml penicillin/streptomycin (Thermo Fisher Scientific, Waltham, MA, USA). Mab2b was purchased from Synaptic Systems, Göttingen, Germany. Following three washing steps in phosphate-buffered saline (PBS, pH 7.4), cells were incubated with goat-anti-mouse-Cy3 secondary antibody again for 1 h at 4°C (dilution of secondary antibody 1:500 in Earle’s minimal essential medium, Dianova, Hamburg Germany). Glass cover slips with pre-labeled GlyRs were transferred into a 24-well plate containing 500 μl of Earle’s minimal essential medium with PKA and PKC modulators (200 nM final concentration each; Forskolin, H-89, PMA, Staurosporine aglycon, all from Sigma-Aldrich Chemie GmbH, Munich, Germany). Cells were incubated with modulators for 15 min to allow receptor internalization. Time point zero (0 min) was taken as a negative internalization control to ensure health of HEK293 cells (pre-labeling of GlyRs at 4°C inhibits internalization). Following incubation with PKA and PKC modulators, cells were stained with a second secondary antibody (donkey-anti-goat-Dyelight488, 1:250 in Earle’s minimal essential medium, Dianova, Hamburg, Germany) for 30 min at 4°C to label receptor retained at the cellular surface. Cells were fixed with 4% paraformaldehyde, 4% sucrose in PBS for 15 min at 4°C, washed three times in PBS and stained with 4′,6-diamidino-2-phenylindole (DAPI, Thermo Fisher Scientific, Waltham, MA, USA) to label the cell nuclei for 5 min at 4°C (1:5000 in PBS). Coverslips were washed again with PBS and Aqua Dest and mounted on glass slides using mowiol.

### Confocal Microscopy, Image Acquisition

The images were obtained using an inverted Olympus IX81 microscope equipped with an Olympus FV1000 confocal laser scanning system, a FVD10 SPD spectral detector and diode lasers of 495 nm (Alexa488) and 550 nm (Cy3; Olympus, Tokyo, Japan), and an Olympus UPLSAPO 60× (oil, numerical aperture: 1.35) objective. Adobe Photoshop (Adobe, San Jose, CA, USA) or ImageJ (1.51)/Fiji^1^ were used for image analysis.

### Electrophysiological Recordings and Data Analysis

Whole-cell recording experiments were performed using a HEKA EPC10 amplifier (HEKA Electronics, Lambrecht, Germany) controlled by Pulse software (HEKA). Recording pipettes were pulled from borosilicate glass (World Precision Instruments, Berlin, Germany) using a Sutter P-97 horizontal puller (Sutter, Novato, CA, USA). Pipettes routinely had an open tip resistance of 2–4 MΩ. HEK293 cells had a membrane capacitance of 3–7 pF. Series resistance, as well as slow and fast capacitance were compensated using the EPC10 circuitry. The different protein kinase modulators were dissolved in intracellular buffer at a concentration of 0.5 μM. For studies of concentration- and time-dependence, kinase modulators were used at concentrations of 0.1 μM and 0.25 μM. Current responses were measured at room temperature (21–23°C) at a holding potential of −50 mV. Solutions were applied using an Octaflow system (NPI electronics, Tamm, Germany), where cells were bathed in a laminar flow of buffer, giving a time resolution for solution exchange and re-equilibration of about 100 ms. The external buffer consisted of 135 mM NaCl, 5.5 mM KCl, 2 mM CaCl_2_, 1.0 mM MgCl_2_ and 10 mM Hepes (pH adjusted to 7.4 with NaOH); the internal buffer was 140 mM CsCl, 1.0 mM CaCl_2_, 2.0 mM MgCl_2_, 5.0 mM EGTA and 10 mM Hepes (pH adjusted to 7.2 with CsOH). Dose response data was fitted to the Hill equation

$$\frac{I_{\text{glycine}}}{I_{\text{sat}}}=\;\frac{{\left[Glycine\right]}^{\text{nHill}}}{{\left[Glycine\right]}^{\text{nHill}}+EC_{\text{50}}{}^{\text{nHill}}}$$

using a nonlinear algorithm in Microcal Origin. Here, I_glycine_ is the current amplitude at a given glycine concentration, I_sat_ is the maximum current amplitude at saturating concentrations of glycine, EC_50_ is the glycine concentration at half-maximal current responses, and nHill is the Hill coefficient. Currents from each individual cell were normalized to the maximum response at saturating glycine concentrations. Significance of differences between EC_50_ values were determined using one-way ANOVA with *p* ≤ 0.05 (*) and *p* ≤ 0.01 (**) taken as significant. In all experiments, EC_50_ values were determined for each individual cell from a non-linear fit of dose response data to the logistic equation. An unweighted average ± standard error (SEM) was calculated from all individual EC_50_ values, without considering the fitting errors.

For time course analysis, a series of EC_50_ data was recorded from each cell. The first concentration-response curve, defined as *t* = 0, was recorded immediately after establishing the whole-cell configuration and positioning the cell in front of the delivery tube. The dead time did not exceed 2 min. Further recordings were made at *t* = 3 min, *t* = 6 min and *t* = 10 min (not attained with all cells). Time-independent recordings were taken at *t* = 0, and values of EC_50_ and I_max_ in the presence of PKA- or PKC modulators were normalized to control (no modulator). For time course analysis, responses were normalized to the control value at *t* = 0. Patch-clamp experiments were carried out on different dates and using different batches of HEK 293 cells. Since control EC_50_ values varied between different days of plating and between batches of cells, measurements with kinase modulators were always compared to control measurements recorded on the same day and from the same batch of cells. Thus, while individual EC_50_ values varied between cells, any changes of EC_50_ induced by kinase modulators could robustly be observed and quantified.

## Results

**Figure 2:**
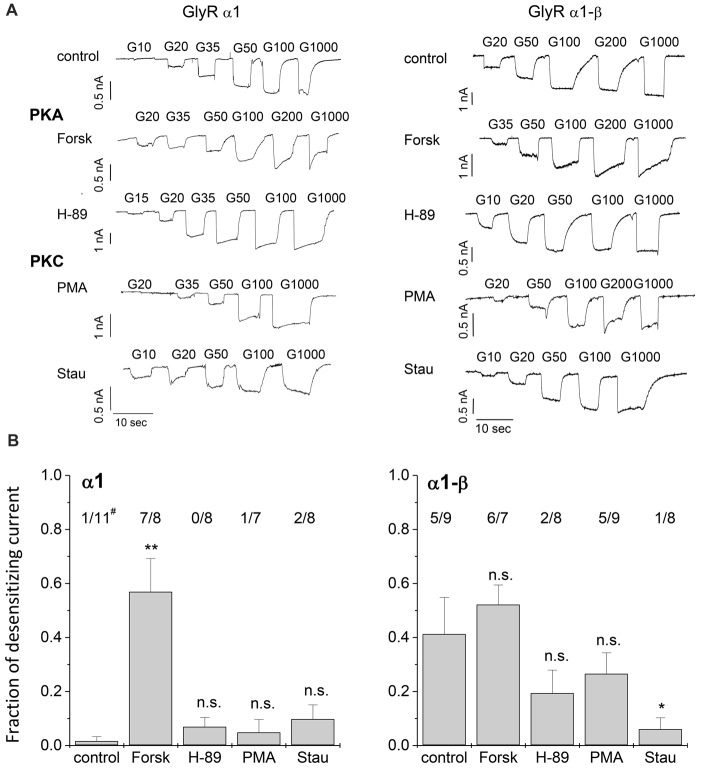
Whole-cell current recordings. **(A)** Glycine induced currents in HEK293 cells transfected with GlyR α1 or GlyR α1-β in absence of protein kinase modulators (control), with protein kinase A (PKA) activator Forskolin (Forsk) and inhibitor H-89, as well as PKC activator phorbol-12-myristate-13-acetate (PMA) and inhibitor Staurosporine aglycon (Stau). Modulators were added to the intracellular buffer at a concentration of 0.5 μM. **(B)** Desensitization behavior of each single cell was studied. Fraction of desensitization was calculated by dividing I_desen_/I_max_. The number of cells showing desensitizing currents (I_desen_/I_max_ > 0.25) out of the total number of tested cells is shown above each column (#). Significance levels (one-way ANOVA) are indicated (*p < 0.05, **p < 0.01, n.s., not significant).

**Figure 3:**
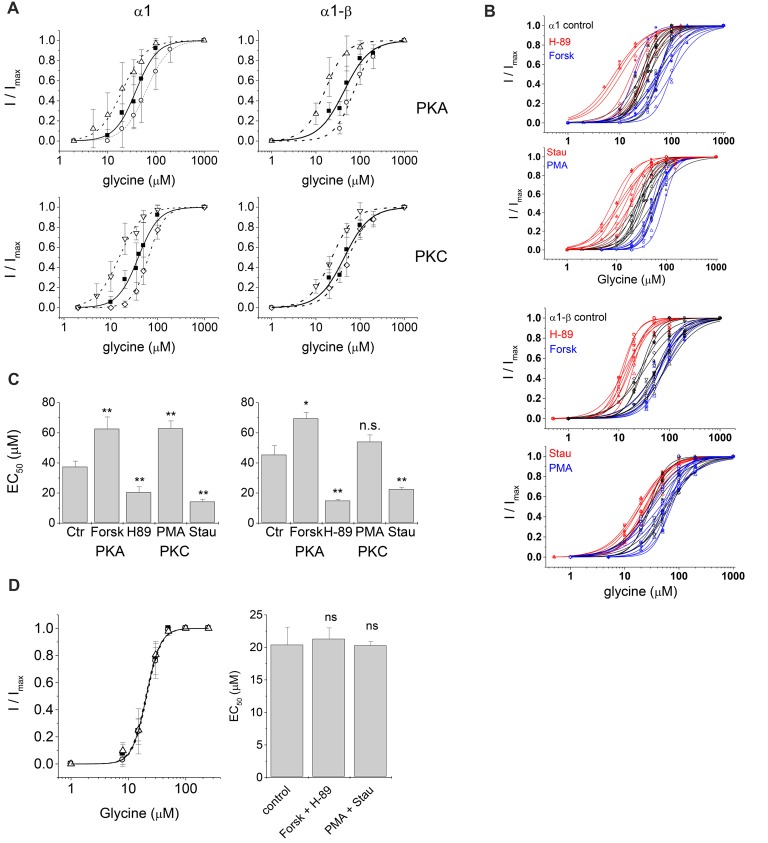
Electrophysiological characterization of glycinergic currents upon protein kinase modulation. Dose-response curves of glycine induced currents in HEK293 cells transfected with GlyR α1 or GlyR α1-β in absence (control) and presence of kinase modulators applied in the intracellular buffer. **(A)** Control: solid squares, solid line; 0.5 μM Forskolin (Forsk): open circle, dashed line; 0.5 μM H-89: open up triangle, dash-dotted line; 0.5 μM PMA: open diamond, dashed line; 0.5 μM Staurosporine aglycon (Stau): open down triangle, dash-dotted line. All curves were fitted according to the Hill equation. Curves show a rightward shift of the dose-response curve in case of activation due to Forskolin or PMA treatment and a left shift in case of inhibition due to H-89 or Staurosporine aglycon treatment. **(B)** Single cell EC_50_ data of control (black), PKA and PKC activator (blue) and PKA and PKC inhibitor (red). **(C)** Summary of whole-cell current characteristics for PKA and PKC modulation. Significance levels for the comparison of EC_50_ (*p*-values, one-way ANOVA) are indicated (*p ≤ 0.05, **p ≤ 0.01, n.s., not significant). All changes in EC_50_ values were significant except PMA treatment for heteromeric α1-β GlyRs. Here we detected an increase in EC_50_ values, however due to the daily variability in EC_50_ values this effect was not statistically significant. **(D)** Simultaneous intracellular application of PKA modulators. Forskolin and H-89, and PKC modulators PMA and Staurosporine aglycon. All modulators were used at a concentration of 0.25 μM. Left panel: concentration-response curves: solid squares: control, no modulators; open circles: Forskolin/H-89; open triangles: PMA/Staurosporine aglycon. Right panel: summary of results in a bar graph. No significant changes from control were observed, indicating that indeed action of PKA- and PKC agonists were counteracted by inhibitors (n.s., not significant).

To investigate the effects of protein kinase modulation on GlyR-mediated currents, α1 and α1-β GlyR subunits were expressed in HEK293 cells and studied using whole-cell patch-clamp recording techniques. Protein kinase activators and inhibitors were added to the intracellular buffer. Control measurements were performed in the absence of modulators (Figure 2). Averaged data from cells expressing GlyR α1 and GlyR α1-β was used to construct dose response curves (Figure 3). Glycine EC_50_ values were determined for each individual cell and then averaged. EC_50_ for glycine was 37.4 ± 3.7 μM (*n* = 11) and 45.4 ± 6.0 μM (*n* = 8) for α1 and α1-β GlyR, respectively (Table 1). GlyR α1-mediated currents showed no or very slow desensitization. Responses from heteromeric α1-β GlyRs, on the other hand, varied from cell to cell, showing both, fast desensitizing (50%) or slow to non-desensitizing currents (Figure 2).

**Table 1:** Summary of electrophysiological data.

			EC_50_ (μM)	nH	*I*_max_ (nA)	Fraction of desensitizing cells	*N*
PKA	Control	α1	37.4 ± 3.7	2.52 ± 0.22	2.19 ± 0.35	0.02 ± 0.02	11
		α1-β	45.4 ± 6.0	1.82 ± 0.16	1.19 ± 0.37	0.41 ± 0.14	8
	Forskolin	α1	62.6 ± 7.9	2.20 ± 0.26	1.20 ± 0.24	0.57 ± 0.12	9
		α1-β	71.5 ± 4.3	1.95 ± 0.14	0.99 ± 0.24	0.52 ± 0.07	7
	H-89	α1	20.5 ± 3.7	2.10 ± 0.27	1.91 ± 0.57	0.07 ± 0.03	8
		α1-β	14.9 ± 1.0	2.31 ± 0.21	1.37 ± 0.28	0.19 ± 0.09	7
PKC	PMA	α1	63.0 ± 5.0	1.70 ± 0.11	2.30 ± 0.74	0.05 ± 0.05	7
		α1-β	54.0 ± 4.6	2.00 ± 0.19	1.26 ± 0.27	0.27 ± 0.08	8
	Staurosporine aglycon	α1	14.3 ± 1.6	1.96 ± 0.17	1.70 ± 0.29	0.10 ± 0.05	8
		α1-β	22.6 ± 1.3	1.82 ± 0.12	1.11 ± 0.20	0.06 ± 0.04	8

*All values are mean ± SEM*.

### Effects of PKA Modulation on GlyR Currents

PKA activator Forskolin, as well as the inhibitor H-89 were added to the intracellular buffer separately, at a concentration of 0.5 μM (Figure 2). Control cells transfected with homomeric α1 subunits were not desensitizing while heteromeric α1-β cells showed desensitization in ~50% of all cells. When H-89 was present in the intracellular buffer, homomeric α1, as well as heteromeric α1-β receptors gave non-desensitizing current responses. Forskolin on the other hand, induced a change to faster desensitization in all cells transfected with either α1 or α1-β GlyR subunits (Figure 2B). Considering channel activation, internal Forskolin treatment gave average EC_50_ values of 62.6 ± 7.9 μM (*n* = 9) for α1 and 71.5 ± 4.3 μM (*n* = 7) for α1-β GlyRs (Figures 3A,B). Hence, Forskolin application caused a significant increase in the EC_50_ value, while H-89 decreased EC_50_ values compared to control giving an EC_50_ value of 20.5 ± 3.7 μM (*n* = 8) for α1 GlyRs and 14.9 ± 1.0 μM (*n* = 7) for heteromeric α1-β receptors, both changes were statistically significant (*p* < 0.05, one-way ANOVA; Figure 3C).

### Effects of PKC Modulation on GlyR-Mediated Currents

Presence of intracellular PMA had no effect on receptor desensitization, in contrast to fast desensitization induced by the PKA activator Forskolin. When Staurosporine aglycon was present glycine induced currents showed slow or no desensitization for both, α1 and α1-β receptors. For comparison, unmodified α1 GlyR was non-desensitizing, while α1-β subunits showed 50% desensitizing cells. Thus, significant differences were only observed for α1-β receptors (Figure 2B). EC_50_ values for glycine were increased after intracellular application of 0.5 μM PMA compared to untreated cells. These changes were statistically significant for α1 receptors, but not for heteromeric α1-β subunits. The EC_50_ values were 63.0 ± 5.0 μM (*n* = 7) and 54.0 ± 4.6 μM (*n* = 8) for α1 GlyRs and α1-β GlyRs, respectively (Figure 3C). The PKC inhibitor Staurosporine aglycon resulted in a decrease of half maximum values of α1 (EC_50_ = 14.3 ± 1.6 μM, *n* = 8), and α1-β receptors (EC_50_ = 22.6 ± 1.3 μM, *n* = 8; Table 1, Figure 3), both changes being statistically significant (*p* < 0.01).

We noticed that PKA and PKC inhibitors weakened the HEK293 cells more than the activators. When the intracellular concentration of H-89 or Staurosporine aglycon was raised to 1 μM, cells did not survive.

To test if the observed shifts in GlyR responses were indeed due to changes in PKA- and PKC modulation, we performed a separate experiment where kinase agonist and antagonist were applied simultaneously (Figure 3D). To avoid cell damage, this experiment was performed using 0.25 μM of each drug, Forskolin and H-89 (PKA), or PMA and Staurosporine aglycon (PKC). EC_50_ of GlyR control currents (no modulator) was 20.4 ± 2.7 μM (*n* = 3), in presence of 0.25 μM Forskolin and 0.25 μM H-89, EC_50_ was 21.3 ± 1.7 μM (*n* = 3), while in presence of 0.25 μM PMA and 0.25 μM Staurosporine aglycon, EC_50_ = 20.3 ± 0.6 μM (*n* = 3) was found, all changes were not significant (*p* > 0.1, one-way ANOVA). Thus, any shift induced by the PKA-activator Forskolin, or the PKC-activator PMA was counteracted by the corresponding antagonists, H-89 and Staurosporine aglycon, respectively.

### Concentration-Dependence of PKA- and PKC-Modulation of GlyR Activity

**Figure 4:**
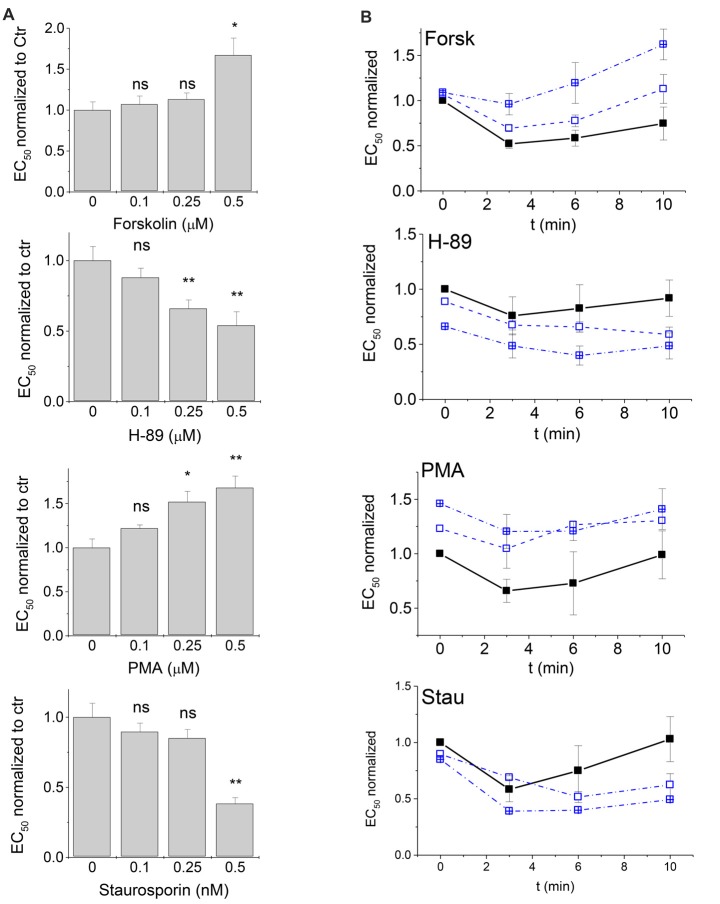
Time- and concentration-dependence of PKA and PKC modulation. PKA- and PKC-modulators were applied in the patch clamp pipette at concentrations of 0.1 μM, 0.25 μM and 0.5 μM (data at 0.5 μM are the same as in Figures 2, 3). **(A)** EC_50_ values of the first measurement after entering the patch (*t* = 0) were compared. All EC_50_ values in the presence of modulators were normalized to control (intracellular buffer without additions) that were measured in the same time period. **(B)** Changes of glycine EC_50_ upon PKA and PKC modulation were followed up to 10 min after the first recording (see “Materials and Methods” section) for modulator concentrations of 0 μM (control), 0.1 μM and 0.25 μM. Solid squares, solid line: control; open squares, dashed line: 0.1 μM modulator; open crossed squares, dash-dotted line: 0.25 μM modulator. EC_50_ values at *t* = 0 were normalized to control in the absence of modulators. EC_50_ values after 3, 6 and 10 min were determined and normalized to the EC_50_ observed for the same modulator at *t* = 0. Each time point was measured from 3–5 cells and normalized currents were averaged. Statistical significance is indicated (*p < 0.05; **p < 0.01; n.s., not significant). Note that EC_50_ changes over time even in absence of modulators. Addition of PKA- and PKC modulators induces a significant additional shift of concentration-response characteristics.

There was only a narrow window to study the concentration-dependence of protein kinase modulation on glycinergic currents, since concentrations larger than 0.5 μM were not tolerated by the cells. We chose values between 0.1 μM and 0.5 μM of each modulator. The effects of all four tested kinase modulators were indeed concentration dependent. The observed EC_50_ values were normalized to control. Shifts at the lowest concentration of modulators (0.1 μM) were not significant, but did show a clear trend, while at 0.25 μM of modulator, significant effects were observed for H-89 and PMA, but not for Forskolin or Staurosporine aglycon (Figure 4A).

### Time Course of GlyR-Mediated Currents Responses After PKA- and PKA-Modulation

To identify the time-dependence of any changes in EC_50_ of GlyR-mediated currents, we performed a series of experiments where EC_50_ was recorded from the same cell after *t* = 0 min (i.e., immediately after patching and positioning the cell), and again after *t* = 3, 6 and 10 min using 0.1 μM and 0.25 μM of each kinase modulator in the intracellular pipette (Figure 4B). Responses from each cell were normalized first to control values at *t* = 0 (see Figure 4A) to compensate for the 2-min dead time between establishing whole cell configuration (start of modulator activity) and the first recording at *t* = 0. For each cell, EC_50_ values at *t* = 3, 6 and 10 min were then normalized to the value at *t* = 0. Time-dependence revealed two simultaneous effects: (i) a time-dependent change in receptor activation where EC_50_ values were reduced between 3 min and 6 min after the first measurement. This effect was independent of the absence or presence of kinase modulators; and (ii) distinct effects of the four protein kinase modulators on GlyR EC_50_ that were reproducibly observed and were additive to the first effect. Presence of the PKA-activator Forskolin and the PKC-activator PMA shifted EC_50_ towards higher values, while PKA antagonist H-89, and the PKC-antagonist Staurosporine aglycon shifted EC_50_ towards lower values (Figure 4B). Thus, time course analysis robustly confirmed the effects of protein kinase modulators on GlyR current responses. It is noted that the effects of lower concentrations (0.1 μM and 0.25 μM) became more pronounced at longer time of application (Figure 4B).

**Figure 5:**
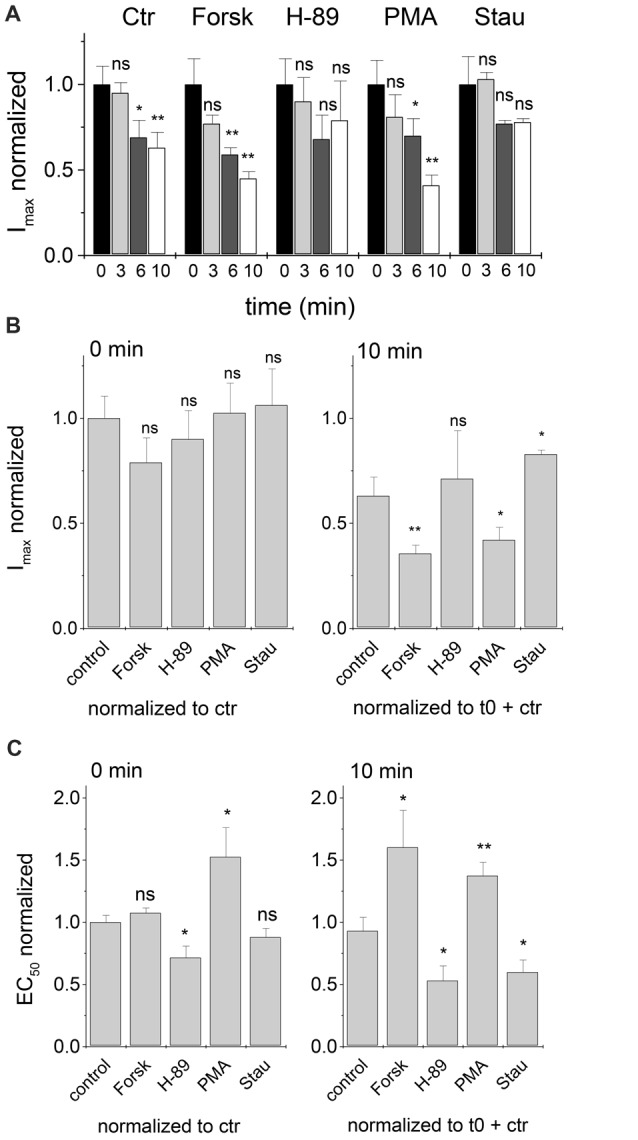
Time dependent development of I_max_ and EC_50_ after treatment with PKA and PKC modulators. Changes in I_max_ and EC_50_ values were followed for 10 min after the first current recording. Modulator concentrations were 0.25 μM. **(A)** Comparison of I_max_ values of PKA and PKC modulators after 0, 3, 6 and 10 min. I_max_ values were normalized to *t* = 0. **(B)** Start (*t* = 0 min) and end (*t* = 10 min) maximum currents of various modulators were compared to control currents. **(C)** EC_50_ values at *t* = 0 and 10 min were compared to control values. Half-maximum concentrations of glycine in the presence of PKA and PKC modulators at *t* = 0 were normalized to control (no modulation). Additionally, 10 min data points were normalized to the corresponding starting EC_50_ (*t* = 0). Each data point was measured for at least three cells and normalized EC_50_ values were averaged. Statistical significance is indicated (*p < 0.05; **p < 0.01; n.s., not significant).

When maximum currents were followed over a time of 10 min and normalized to the values at *t* = 0, I_max_ was significantly reduced (*p* < 0.05, one-way ANOVA) for control (no modulator), and also in the presence of 0.25 μM intracellular Forskolin or PMA (Figure 5A). In contrast, changes of I_max_ at *t* = 10 min were not significant in the presence of 0.25 μM H-89, or Staurosporine aglycon (Figure 5A).

At *t* = 10 min, I_max_ was reduced relative to the value at *t* = 0 (Figure 5B, right vs. left panel). At *t* = 0 all protein kinase modulators were without significant effect when I_max_ was normalized to control (no modulator; Figure 5B, left panel). When compared to control value at *t* = 10 min, maximum currents were reduced in the presence of kinase agonists and increased in presence of protein kinase antagonists (Figure 5B, right panel).

In case of EC_50_, effects of kinase modulators were apparent even at *t* = 0. Here, kinase agonists Forskolin and PMA increased EC_50_, while kinase antagonists H-89 and Staurosporine aglycon had the opposite effect. Kinase-induced changes were only significant for H-89 and PMA (Figure 5C, left panel). At *t* = 10 min, changes of EC_50_ induced by all PKA- and PKC-agonists and antagonists were significant (Figure 5C, right panel). It is noted that the concomitant changes in I_max_ and EC_50_ may be expected for changes of receptor channel gating, where reduced channel open probability would result in an increase of EC_50_ and lowering of I_max_, and vice versa for increased P_open_ of the channel. Lower values of I_max_ might, however, be due to changes in the number of active receptors on the cell surface. We thus tested whether protein kinase modulation had an effect on receptor density on the transfected cells.

### Cell Surface Expression and Internalization Assay

**Figure 6:**
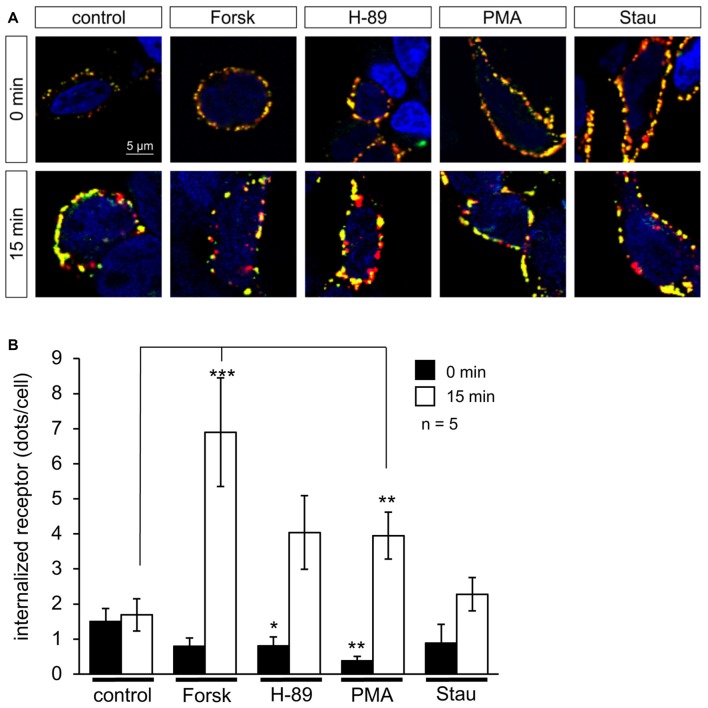
PKA and C activators enhance GlyR internalization. HEK293 cells transfected with GlyRα1-β were pre-labeled with the specific α1 antibody MAb2b and goat-anti-mouse Cy3-coupled secondary antibody, followed by incubation with PKA activator Forskolin (Forsk), PKA inhibitor H-89, PKC activator PMA and PKC inhibitor Staurosporine aglycon (Stau) for 0 min and 15 min in cell culture medium at 37°C and 5% CO_2_. Retained receptors at the surface were double labeled using a donkey-anti-goat-Dyelight488 antibody. **(A)** Representative cells at various time points (0, 15 min) incubated with PKA and PKC activators and inhibitors showing internalized receptor (red dots), and receptor retained at the cellular surface (yellow dots), control always shows untreated cell (UT). White arrows point to internalized receptors. Nuclei (blue) were marked by 4′,6-diamidino-2-phenylindole (DAPI). **(B)** Quantification of internalized receptor (red dots/cell) at two different time points (0 min = black bars; 15 min = white bars). Averages from five independent experiments ± SEM are given, determined from 16 to 51 cells per condition. Significance values (one-way ANOVA) *p < 0.05, **p < 0.01, ***p < 0.001.

Cell surface expression of GlyR α1 homomers and α1-β heteromers was determined by biotinylation of cell surface protein and subsequent discrimination of surface and whole cell protein by Western Blot analysis (data not shown). Prior to protein biotinylation, PKA modulators and PKC modulators were incubated with transfected cells for 16 h. Neither PKA nor PKC modulators had a significant effect on GlyR α1 and GlyR α1-β whole cell or surface expression. Local changes in surface expression might, however, be overlaid by continuous overexpression of GlyRs in transfected HEK293 cells during the incubation period of 16 h. Previously, it has been shown that receptor internalization following PMA treatment starts already within 15 min (Huang et al., 2007). Therefore, we performed short-term receptor internalization experiments using PKA and PKC modulators. To distinguish internalized receptor from newly synthesized receptor protein, GlyRs were fluorescence labeled using an α1-specific antibody at 4°C followed by a period of incubation with PKA modulators (Forskolin and H-89) or PKC modulators (PMA and Staurosporine aglycon) at 37°C to allow receptor internalization. Internalized receptors were imaged (Figure 6A) and quantified (Figure 6B) in comparison to untreated cells. Activators of PKA (Forskolin) and PKC (PMA) significantly enhanced GlyR internalization after 15 min (Forskolin 6.9 ± 1.5 dots/cell, *p* = 9.3E-05; PMA 3.9 ± 0.7 dots/cell, *p* = 0.006; in comparison to untreated cells UT 1.7 ± 0.4 dots/cell). In presence of kinase inhibitors H-89 (PKA) and Staurosporine aglycon (PKC), internalization was lower compared to the corresponding PKA and PKC activators. It is noted, that even in presence of the inhibitors, overall internalization was increased relative to control, but these increases were not significant (H-89: 4.0 ± 1.0 dots/cell, *p* = 0.09; Staurosporine aglycon: 2.3 ± 0.5 dots/cell, *p* = 0.200; Figure 6B).

The observed short-term effects of PKA and PKC modulators on receptor internalization were visible in a similar time frame to that of electrophysiological recordings, indicating that kinase modulators were able to induce fast changes of receptor properties on a time scale of minutes following their application.

## Discussion

Phosphorylation of neuronal receptors is an important mechanism of the regulation of receptor expression and function. In case of the cys-loop receptor family, phosphorylation sites have been mainly mapped to the highly variable TM3–4 loop of the mature protein, where different subunits of the receptor show various phosphorylation sites. Two kinases are mainly involved in phosphorylation of the intracellular loop namely, PKC and cAMP-dependent protein kinase A (PKA). GlyR α1 subunits isolated from rat spinal cord were shown to be phosphorylated at Ser391 by PKC *in vitro*, with the phosphorylated serine residue located within a distinct PKC phosphorylation consensus sequence in the cytoplasmic TM3–4 loop (Figure 1; Ruiz-Gómez et al., 1991). In contrast to PKC, most GlyR α1 subunit isoforms do not contain consensus sites for PKA phosphorylation. One exception is the α1^ins^ splice variant containing an 8-amino acid insert (^326^SPMLNLFQ^333^, numbers referring to the mature protein) in the TM3–4 domain. Here, the first residue of this insert may serve as a phosphorylation site for PKA (Malosio et al., 1991; Lynch, 2004). The α1 sequence used in our study did not contain the insert, thus lacking this potential PKA phosphorylation consensus sequence. Therefore, the effects on homomeric α1 glycinergic currents might be mediated by indirect interactions involving multiple intracellular processes downstream of PKA activation (Vaello et al., 1994). The β-subunit, on the other hand contains a PKA consensus site at position T363 in the TM3–4 domain (Grenningloh et al., 1990). However, it is not yet determined whether the α1^ins^ or the β-subunit sites are directly phosphorylated (Lynch, 2004). The GlyR β-subunit also contains a putative PKC phosphorylation consensus site at position S389 in the TM3-4 domain, whose functional status remains to be confirmed (Grenningloh et al., 1990; Lynch, 2004). Recently, an additional PKC phosphorylation site was identified in the β-subunit (Specht et al., 2011). This phosphorylation site (S403) is located at the end of the TM3–4 and influences the binding affinity between the receptor and gephyrin. Phosphorylation of α3 GlyRs, which alters receptor function and its role of in spinal nociceptive signaling has been well studied and reviewed previously (Harvey et al., 2004; Zeilhofer, 2005; Han et al., 2013).

In the literature, the specific phosphorylation sites and the resulting effects on GlyR function have been explored in different cell types using different techniques, often yielding inconsistent and even contradictory results. This can be attributed to differences in receptor clustering and the presence of cytoskeletal proteins which seem to affect the accessibility of receptor phosphorylation sites for the kinases/phosphatases. Furthermore, it is postulated that these enzymes could exert a direct action on other modulatory proteins that directly interact with the receptor, adding to the observed tissue-specific differences (Lynch, 2004). Some of the contradictory effects of cAMP-dependent phosphorylation as well as protein kinase C may be explained by the differential expression of α1^ins^- and β-subunits throughout the central nervous system (Legendre, 2001). Here, we investigated the role of protein kinases in the modulation of glycinergic transmission. We concentrated on functional aspects using whole-cell patch-clamp technique as well as GlyR expression.

We used established, specific modulators of PKA and PKC. For PKA, we used Forskolin, a diterpene from the Indian Coleus plant (*Plectranthus barbatus*), which activates adenylyl cyclase, leading to an increase of cAMP levels, which in turn increases PKA activity, and H-89, a competitive inhibitor of the ATP binding site of PKA. PKC activation was done using PMA, a structural analog of the natural PKC activator diacyl-glycerol. For PKC inhibition, we used Staurosporine aglycon, a natural indole antibiotic from *Streptomyces staurosporeus*, which acts as competitive inhibitor of ATP binding to PKC.

### PKA Modulation of GlyR-Mediated Currents

The functional effect of PKA activation and inhibition on glycine induced currents was examined in HEK293 cells expressing α1 and α1-β GlyRs. For electrophysiological measurements we applied the modulators in the intracellular medium at concentrations of 0.5 μM, or at 0.1 μM and 0.25 μM when time- and concentration-dependence of kinase modulators were studied in α1 homomeric receptors. Intracellular application of modulators helped to avoid possible direct effects of the modulators on the GlyR as has been reported previously (Wagoner and Pallotta, 1988; Swope et al., 1992) and ensured rapid action of the respective kinases. The PKA activator Forskolin caused a significant increase in half maximum concentration of current activation by glycine for both, α1 and α1-β receptors, but had no significant effect on maximum currents. Forskolin, however, affected desensitization behavior of α1- and α1-β receptors as glycine-mediated currents from cells treated with Forskolin showed faster desensitization than untreated controls. The change to fast desensitization was significant for homomeric α1 receptors, while for α1-β control currents partial desensitization (>10% of desensitizing current) was observed in five out of nine traces, and addition of Forskolin induced no significant changes. The PKA inhibitor H-89, on the other hand, reduced EC_50_ values significantly, this effect being more pronounced for α1-β variants than for monomeric α1 subunits. H-89 had no significant effect on desensitization of homomeric α1 receptors, currents were slow- or non-desensitizing, similar to untreated controls. Glycine-induced α1-β currents, on the other hand, were predominantly non-desensitizing in the presence of H-89, while control currents were both, desensitizing, or non-desensitizing, varying from cell to cell. The considerable variability in the desensitization of neuronal GlyR is known in the literature, but not yet fully understood (Gielen and Corringer, 2018). Reports in the literature on activation and inhibition of GlyR function by PKA are quite contradictory. In periaqueductal gray neurons, Forskolin inhibited I_gly_ potentiation induced by μ-opioid agonist D-Ala 2, N-MePhe4, GlyS-ol-enkephalin, while PKA inhibition by H-89 resulted in a potentiation of I_gly_ (Min et al., 1996). These effects of Forskolin and H-89 are in agreement with our observations. Li and Yang (2001) studied the effect of Forskolin on isolated carp amacrine-like cells, where Forskolin markedly accelerates desensitization of whole cell responses but the effect cannot be blocked by PKA inhibitors like H-89 and H-8. Also, 1,9-dideoxy Forskolin, an inactive analog of Forskolin accelerates the desensitization effectively, indicating that changes in desensitization behavior are not mediated by cAMP-dependent protein kinase. Authors suggest that Forskolin might act directly on an extracellular site of the GlyR (Li and Yang, 2001). Similar direct effects of Forskolin were also reported in intact skeletal muscles, where Forskolin induced enhancement of desensitization of the nAChR in a cAMP-independent manner (Wagoner and Pallotta, 1988). In the present study, Forskolin application was intracellular, thereby excluding the possibility of an extracellular binding site responsible for altered desensitization behavior. Nevertheless, we observed accelerated desensitization after internal Forskolin treatment suggesting that there must be an additional aspect—maybe phosphorylation after all—that is responsible for the observed changes in desensitization. Gentet and Clements studied phosphorylation on GlyR α1 expressed in HEK293 cells (Gentet and Clements, 2002). They observed that phosphorylation accelerated desensitization of glycine induced currents, in agreement with our data. A further phosphorylation study on ^3^H-glycine and ^3^H-strychnine specific binding to GlyRs in intact isolated frog retinas showed that specific binding of both radioligands was reduced 30%–90% after activation of PKA with 10 μM Forskolin (Salceda and Aguirre-Ramirez, 2005), in good agreement with reduced receptor activity after PKA treatment observed in this study. In case of α2 and α3 receptors, Ser-346, located within the PKA consensus sequence (R-E-S-R, not present in α1) of the GlyR α3 intracellular loop has proven to be an essential target for prostaglandin-E(2)-mediated phosphorylation. Stimulation of the prostaglandin E2 receptor by PGE2 results in a cAMP-mediated activation of PKA. Phosphorylation of Ser-346 slowed desensitization and decreased glycinergic currents in HEK293 cells expressing GlyR α3 and α2 subunits (Heindl et al., 2007).

GlyR modulation through PKA was described in other expression systems. Potentiation of chloride currents after PKA activation was observed in spinal trigeminal neurons (Song and Huang, 1990), *Xenopus* oocytes expressing α1 GlyRs (Vaello et al., 1994), and trigeminal neurons (Gu and Huang, 1998). Ren et al. (1998) studied cAMP-dependent PKA modulation of glycine activated chloride currents in single neurons from rat ventral tegmental area in whole cell patch clamp technique. The current induced by small glycine concentrations (3–10 μM glycine) increased spontaneously after internal application of Mg-ATP. When H-89 was added to the pipette solution, ATP induced run-up of the glycine current was blocked (Ren et al., 1998). The contradictory results reported in the literature may likely be a consequence of differential expression of GlyR subunits throughout the central nervous system (Legendre, 2001).

### PKC Modulation of GlyR-Mediated Currents

Activation of PKC by PMA resulted in significantly increased EC_50_ values for glycine in HEK293 cells expressing α1 receptors compared to untreated controls. The same tendency was seen in cells expressing heteromeric α1-β subunits. Here, changes were not statistically significant but the trend was similar to that seen in homomeric receptors. PKA and PKC inhibitors H-89 and Staurosporine aglycon, respectively, reduced GlyR EC_50_, changes were significant for both α1 and α1-β subunits. Similar to PKA modulators, we did not observe any significant changes in maximum currents. In homomeric α1 receptors slow desensitization was observed in case of control cells as well as cells treated with PMA. In α1-β receptors we observed non-desensitizing as well as fast desensitizing currents for both, control and PMA treated cells, indicating that PKC activation by PMA had no effect on desensitization. Staurosporine aglycon treatment resulted in non- or slowly desensitizing currents for both, α1 and α1-β receptors. In case of α1 receptors this was unchanged from controls. For cells expressing α1-β receptors, on the other hand, changes in desensitization behavior were statistically significant. While control cells showed heterogeneous desensitization behavior that varied from cell to cell, all cells treated with Staurosporine aglycon gave non-desensitizing currents. Our observation that receptor function is reduced upon PKC activation and increased upon PKC inhibition has been reported previously. GlyR expressed in *Xenopus* oocytes injected with mRNA isolated from nervous tissue showed decreased glycinergic currents after PMA-induced activation indicating that the PKC system is involved in negative regulation of the GlyR channel (Vaello et al., 1994). Reduced responses were also reported when homomeric α1 GlyRs expressed in *Xenopus* oocytes were activated by 4-beta-PMA. In the presence of Staurosporine aglycon the effect was inhibited. 4-alpha-PMA, a poor PKC stimulant, did not affect the glycine currents, indicating that the PKC system is involved in negative regulation of the GlyR channels (Uchiyama et al., 1994). PKC activation by PMA was found to reduce the amplitude of glycine currents, in agreement with our results (Tapia et al., 1997). The same study, however, reports that PKA activation potentiates glycine induced currents, which is in contrast to our findings (Tapia et al., 1997).

Recently, the effects of receptor phosphorylation on specific binding of ^3^H-Gly and ^3^H-Str were investigated. The binding of both radioligands was remarkably reduced after phorbol ester activation in isolated frog retinas (Salceda and Aguirre-Ramirez, 2005), in agreement with our findings. In a similar study, specific radioligand binding after PKA or PKC activation was decreased by 60%–85%, an effect that could be blocked by specific kinase inhibitors (Velázquez-Flores and Salceda, 2011). The effect of temperature on PKC modulation was investigated in *Xenopus* oocytes and HEK293 cells (Machu et al., 2006). PMA produced a large, reproducible decrease in the peak amplitude of GABA- and Gly-gated currents in *oocytes*. In contrast, PMA was ineffective in modulating these receptors at room temperature in HEK293 cells. When electrophysiological experiments were performed at 35°C in HEK293 cells, PMA decreased the function of these receptors. The authors claim that the temperature is an important variable when studies are performed in HEK293 cells expressing GlyRs and could be a reason for contradicting results in former studies (Machu et al., 2006). Enhancement of glycinergic currents upon PKC activation was observed in rat sacral dorsal commissural neurons (Xu et al., 1996), hippocampal neurons (Schönrock and Bormann, 1995) and trigeminal neurons (Gu and Huang, 1998). In summary, neuronal cell type and location seem to be important parameters that might cause controversial observations after PKC or PKA modulation (Lynch, 2004). Furthermore, temperature was suggested as a relevant parameter for PKC modulation of ligand-gated ion channels (Machu et al., 2006).

### Concentration Dependence and Time Course of PKA- and PKC-Modulation of GlyR-Mediated Currents

We noticed that the effects of PKA- and PKC-modulators on GlyR currents were concentration-dependent. At lower concentrations of kinase modulators (0.1 and 0.25 μM) changes were all significant after 10 min of application. Changes of EC_50_ were robust and reproducible in all experimental settings. Time-dependent changes in I_max_ were only apparent after 10 min of application of PKA- and PKC-modulators (0.25 μM). The time dependent reduction of I_max_ was opposed by PKA and PKC inhibitors. A concomitant shift of EC_50_ towards lower values and increase of I_max_ would be consistent with increased channel open probability, i.e., more effective gating (Colquhoun, 1998). While receptor function is clearly affected by the absence or presence of PKA- and PKC modulators, more experiments are required to identify this as a gating effect.

The effects of PKA- and PKC-agonists (Forskolin and PMA, respectively) on GlyR currents could be offset by the addition of the corresponding antagonists, H-89 (PKA) and Staurosporine aglycon (PKC).

### PKA- and PKC-Modulation of GlyR Internalization

No changes in receptor surface expression were obvious after long-term (>1 h) incubation with PKA and PKC modulators. In this situation, the effects of a single application of kinase modulators will be counterbalanced by the general turnover of receptor protein. On a shorter time scale, distinct effects of PKA and PKC modulation could be observed. Activation of either PKA or PKC by Forskolin or H-89 treatment induced enhanced receptor internalization after short-term treatment (15 min), while kinase inhibitors H-89 and Staurosporine aglycon showed no significant effects. These findings are in agreement with electrophysiological data: when PKA and PKC activators were present in the intracellular buffer, a modulation of GlyR currents was detected within ~10 min.

Evidence for the involvement of protein kinases—especially PKC—in the regulation of ion channel surface expression and clustering was described previously for GlyRs, as well as other members of the ligand-gated ion channel family. Huang et al. (2007) showed that endocytosis of the GlyR in HEK293 cells expressing GlyRα1 is mediated by PKC activation and that it is dynamin-dependent. Further evidence that supports the involvement of PKC in GlyR surface expression was a set of immunofluorescent studies done in spinal cord neurons. Labeling studies were directed towards PKC and showed a redistribution of the fluorescent signal towards the cell periphery upon PMA treatment, meaning that PKC is recruited towards the cell periphery to start mediating endocytosis (Velázquez-Flores and Salceda, 2011). Along with reduced specific radioligand binding by 60%–85% after PKA and PKC activation, Velázquez-Flores and Salceda (2011) observed reduced GlyR expression in the plasma membrane by about 50% which was consistent with an increase of the receptor in the microsomal fraction when PKA was activated. The collective data indicates increased receptor endocytosis and reduced cell surface expression as a result of protein kinase activation, suggesting that PKC plays an important role in the balance between endocytosed and surface expressed receptors. Reduced cell surface expression as a consequence of PKC activation was also shown for other ligand-gated ion channels like recombinantly expressed human rho1 GABA_C_ receptors (Filippova et al., 1999), and recombinant GABA_A_ receptors (Connolly et al., 1999). Similar studies on NMDA receptors NR1 and NR2 in cultured hippocampal neurons showed opposite results. Here, 40%–50% of total NR1 immunoreactivity is found at the cell surface, as compared to more than 90% of total NR2B immunoreactivity while metabolic labeling of the cultures with [^32^P] revealed that NR2 subunits are highly phosphorylated under basal conditions, whereas basal phosphorylation of NR1 subunits is barely detectable (Hall and Soderling, 1997). Internalization of GABA_A_ receptors in primary cultures of cerebral cortical neurons was increased upon PKC activation, as demonstrated by limited chymotrypsin digestion assays (Herring et al., 2005), an observation that is in agreement with our data for the expression of heteromeric α1-β GlyRs in HEK293 cells. It should be noted that GlyR retrograde trafficking might be different in neurons where the cytoskeletal anchoring protein gephyrin is cotransported together with the GlyR complex enabling GlyR synaptic integration at postsynaptic membranes. Recent evidence from experiments on murine hippocampal neurons have shown that gephyrin is itself phosphorylated, which allows subsequent interaction with different scaffolding proteins (Zita et al., 2007). These neuron-specific interactions are expected to be important for GlyR function and surface clustering.

Our findings show that activation of PKA and PKC reduces GlyR-mediated currents in a recombinant system. Inhibition of both kinases has the opposite effect. These effects are concentration-dependent and occur within minutes of application. On the same time scale of GlyR current modification, PKA and PKC activators increase internalization of the receptor, while their corresponding inhibitors have an opposite effect. Thus, GlyR phosphorylation is an important physiological regulatory mechanism that is involved in the fine-tuning of expression, trafficking and ion channel function of GlyRs.

## Author Contributions

UB, LMB, DJ and VR performed experiments. UB, CV and H-GB analyzed data. UB, CV and H-GB wrote the manuscript. UB, C-MB, CV and H-GB devised the study.

## Conflict of Interest Statement

The authors declare that the research was conducted in the absence of any commercial or financial relationships that could be construed as a potential conflict of interest.

## Footnotes

^1^https://imagej.net/ImageJ
